# Curriculum-based humanoid robot identification using large-scale human motion database

**DOI:** 10.3389/frobt.2023.1282299

**Published:** 2023-11-30

**Authors:** Sunhwi Kang, Koji Ishihara, Norikazu Sugimoto, Jun Morimoto

**Affiliations:** ^1^ Department of Brain Robot Interface, Computational Neuroscience Laboratories, Advanced Telecommunications Research Institute International (ATR), Kyoto, Japan; ^2^ Graduate School of Informatics, Kyoto University, Kyoto, Japan

**Keywords:** human motion database, humanoid robots, motion retargeting, system identification, dynamics model

## Abstract

Identifying an accurate dynamics model remains challenging for humanoid robots. The difficulty is mainly due to the following two points. First, a good initial model is required to evaluate the feasibility of motions for data acquisition. Second, a highly nonlinear optimization problem needs to be solved to design movements to acquire the identification data. To cope with the first point, in this paper, we propose a curriculum of identification to gradually learn an accurate dynamics model from an unreliable initial model. For the second point, we propose using a large-scale human motion database to efficiently design the humanoid movements for the parameter identification. The contribution of our study is developing a humanoid identification method that does not require the good initial model and does not need to solve the highly nonlinear optimization problem. We showed that our curriculum-based approach was able to more efficiently identify humanoid model parameters than a method that just randomly picked reference motions for identification. We evaluated our proposed method in a simulation experiment and demonstrated that our curriculum was led to obtain a wide variety of motion data for efficient parameter estimation. Consequently, our approach successfully identified an accurate model of an 18-DoF, simulated upper-body humanoid robot.

## 1 Introduction

Due to the nonlinear nature of robotic systems, leveraging accurate models of their dynamics is crucial for fast and precise motion control.

System identification is a methodology that estimates parameters of a dynamical model. For robot systems, the inertial parameters of each link (mass, center of mass position, and inertia matrix) can be estimated from the measurement data of motion trajectories (e.g., joint angles, angular velocities, and torques). Identification methods for robot manipulators have been studied for decades (Mayeda et al., 1984; Atkeson et al., 1986) and applied to complicated multi-degree-of-freedom (DoF) robots, such as humanoid robots (Ayusawa et al., 2008; Mistry et al., 2009). However, identifying reliable inertial parameters remains challenging for humanoid robots.

To estimate reliable inertial parameters, a wide variety of measurement data is required to excite the robot’s dynamics. For robot manipulators, such measurement data can be sampled by designing appropriate reference trajectories by optimization and generating them on the robots (Gautier and Khalil, 1992; Presse and Gautier, 1993). For humanoid robots, however, designing reference trajectories is difficult. Although some strategies have been developed to design robot reference trajectories (Bonnet et al., 2016; Ayusawa et al., 2017), a good model is necessary to consider the constraints of balance of a humanoid robot to generate reference trajectories without falling over. Optimization can also be intractable since the balance constraints are imposed by nonlinear inequality constraints.

To avoid the balance problem, Bonnet et al. (2018) proposed to use a rigid harness to restrain a humanoid robot’s body. Since the harness obviated the risk of falling over, the balance constraints were not necessary. However, the above approach was not able to identify some inertial parameters since the link was fixed by the harness and could not freely move. Therefore, a good initial model remains necessary, and the highly nonlinear optimization problem must be solved to estimate reliable inertial parameters for all links.

Alternatively reference trajectories which ensure the stability of the robots could be chosen from a list of prepared motion candidates (Venture et al., 2009; Jovic et al., 2015). Recently, Aller et al. (2021) proposed to use a large set of static poses for precise estimation of the mass and the center of mass position of each link. Since such static motions are less sensitive to acceleration, it is easy to control balance and the method does not require a highly accurate model. However, the above approach was not able to estimate the moment of inertia due to the lack of dynamic motions. Therefore, it is necessary to derive an accurate initial model so that the robots can perform not only static but also dynamic motions.

To overcome this problem, we propose a curriculum-based identification approach. Our curriculum first provides conservative reference motions that can be generated with an unreliable model. As identification progresses, the curriculum offers more aggressive reference motions so that the robot’s dynamics are fully excited. The initial unreliable model is iteratively updated with the measurement data obtained by generating reference motions. Therefore, since an accurate dynamics model can be learned gradually from an unreliable initial model, a good initial model is unnecessary. Here, a curriculum is represented as a sequence of reference motions for efficient humanoid model identification.

In our proposed method, the curriculum needs to provide a wide variety of reference trajectories, including not only static but also dynamic motions. We utilized captured human motions to obtain such data. Due to the recent development of large human motion databases (Mandery et al., 2015; 2016; Krebs et al., 2021), they now contain a wide variety of motion trajectories. By selecting appropriate human motions from databases and transforming the motion data into the robot’s data, we can easily obtain various reference trajectories. Therefore, our method does not need to solve the highly nonlinear optimization problem to design references. We especially use a recently available database that includes a wide variety of upper-body behaviors of humans (Krebs et al., 2021) to obtain the reference trajectories for an upper-limb humanoid robot.

**FIGURE 1:**
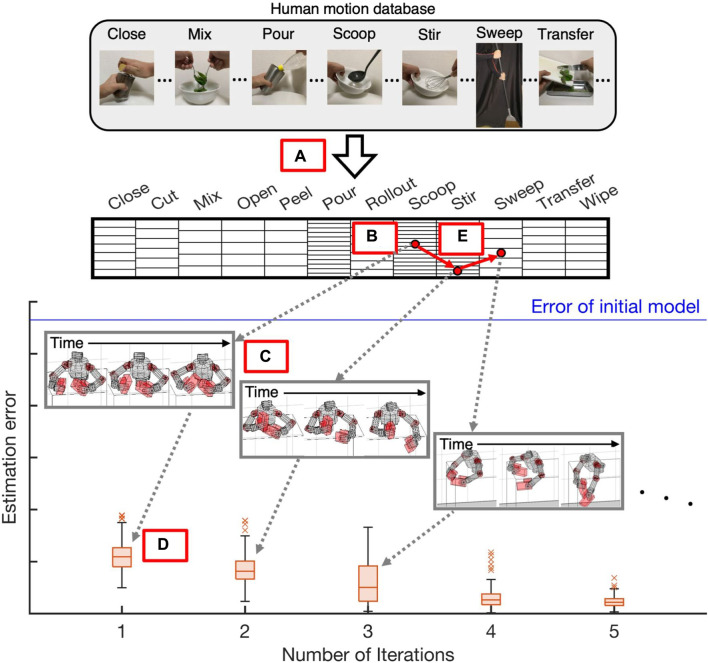
Schematic diagram of proposed identification method: Our method constructs identification curriculum using large-scale human motion database (Krebs et al., 2021) to estimate a dynamics model of humanoid robot from unreliable initial model without solving complex optimization problems: **(A)** Transform human motion database into robot data. **(B)** Search for generable and effective reference motions. **(C)** Provide selected reference and generation to obtain measurement data. **(D)** Estimate new inertial parameters with measurement data. **(E)** Proceed to next iteration and search for new reference motion.

A schematic diagram of our identification curriculum is shown in Figure 1. We first convert the trajectories in the human database into those of the robot to construct a dataset of robot reference motions (Figure 1A). We next search for a generable and effective reference motion for parameter identification through the dataset (Figure 1B). The selected reference is provided to the robot and generated to obtain measurement data (Figure 1C). Finally, the new inertial parameters are estimated with the measured motion trajectories (Figure 1D). The robot can generate more aggressive motions from the next iteration since the model has become more reliable. Thus, in the next iteration such an aggressive motion is selected and provided from the dataset (Figure 1E). This iterative process is repeated until an accurate dynamics model is identified.

The following are the contributions of this study:

• We proposed a curriculum-based humanoid robot identification method that can be adopted even without access to a good initial robot model. Although quasi-static motions are selected with the inaccurate initial model in the first identification step, more aggressive motions become generable from the next step of the iterative identification since the model’s accuracy is gradually improved by the previous iterations.
• In our proposed method, by using large-scale human motion data, the optimization process was greatly simplified for deriving appropriate movement data for system identification than conventional humanoid identification methods. We derived reference data by simply selecting the most suitable data for efficient parameter identification from a large-scale dataset. Note that the most suitable data means one of the candidate motions which can estimate the most reliable parameters. The optimization process to select the suitable data is, in fact, much less computationally intensive than directly solving complex optimization problems considered in the conventional approaches.
• We empirically showed that our identification method can efficiently find the parameters of a humanoid robot model. To identify an accurate model, the data sampling order is significantly important since it affects the sensitivity of the identification results to torque output errors. Through our simulated experiments, we confirmed that our method properly derived the suitable identification sequence and showed that our curriculum-based method achieved efficient parameter estimation.

The paper is organized as follows. Section 2 introduces related studies. Section 3 gives preliminaries on system identification. In Section 4, we describe our curriculum-based identification method for a humanoid robot, and Section 5 shows the results of curriculum-based learning. In Section 6, we summarize and discuss future works.

## 2 Related works

Identification methods for humanoid robots have been developed for over a decade, demonstrating that the inertial parameters can be identified with limited sensor measurements (Ayusawa et al., 2008; Mistry et al., 2009). The physical consistency of inertial parameters can be maintained by an optimization approach (Jovic et al., 2016).

However, the identification of reliable inertial parameters remains challenging for humanoid robots. The strategies that design robot reference trajectories require good initial models to incorporate balance constraints (Baelemans et al., 2013; Bonnet et al., 2016). Although initial models can be obtained from computer-aided-design (CAD) software, the models are often insufficient since a CAD model cannot capture every dynamical effect of the robot (Swevers et al., 2007). Our proposed approach, on the other hand, can estimate reliable inertial parameters without a good initial model using the identification’s curriculum.

A human motion capture database was used for system identification to estimate a human dynamics model (Venture et al., 2009). Human motion data can be utilized to identify a humanoid robot by converting human motion trajectories into those of a robot (Yamane, 2011). However, if the database is small-scale and contains a limited range of motion data, selecting reference trajectories that fully excite the system is difficult (Bonnet et al., 2016). Therefore, we leveraged a large-scale human motion database and show that the dynamics of a humanoid robot are successfully excited since various reference trajectories can be provided from such databases.

To the best of our knowledge, ours is the first work that creates a curriculum of system identification for a humanoid robot. Curriculum-based learning has been used to learn control policies for humanoid robots (e.g. (Karpathy and Van De Panne, 2012; Rodriguez and Behnke, 2021)). We demonstrate that curriculum-based learning is also useful for the identification of a humanoid robot.

Recently, neural-network-based identification methods have been explored for legged robots (Hwangbo et al., 2019; Yu et al., 2019). The robot’s dynamics were modeled with neural networks, and networks parameters were estimated. These studies focused on identifying task-specific dynamics models that did not generalize across multiple motions. Our proposed approach, on the other hand, can identify a non-task-specific model by estimating the physical parameters from a wide variety of measurement data. In the last part of the experiment (Section 5), we demonstrate that our method can obtain more reliable model than the task-specific models.

## 3 Preliminaries on system identification

### 3.1 Dynamics model

In this paper, we address the following dynamics model of a robot system:

$$M\left(\theta \right)\ddot{\theta }+c\left(\theta ,\dot{\theta }\right)+g\left(\theta \right)+v\left(\dot{\theta }\right)=\tau ,$$ (1)

where ***θ***, $\dot{\theta }$ , $\ddot{\theta }$ , and ***τ*** are respectively the vectors of the joint angles, the angular velocities, the angular accelerations, and the joint torques. The inertia matrix is denoted as ***M***, and ***c*** is the centrifugal and Coriolis forces. ***g*** is the gravitational force, and ***v*** is the friction.

### 3.2 Identification process

An identification method estimates the so-called base parameters, which are the minimal identifiable set of the robot’s inertial parameters. They comprise the mass, the CoM position, and the independent inertia tensor coefficients of each link. These parameters are estimated using the fact that the equation of motion 1) can be written in a linear form with respect to base parameter vector ***w*** ∈ ***R*** ^*D*^ (Kawasaki et al., 1991):

$$\phi \left(\theta ,\dot{\theta },\ddot{\theta }\right)w=\tau ,$$ (2)

where ***ϕ*** is called the regressor matrix for the base parameter. If we obtain *T* time-step measurement data of angle trajectories, their velocities and accelerations $Q\equiv \left\{\theta_{1\to T},{\dot{\theta }}_{1\to T},{\ddot{\theta }}_{1\to T}\right\}$ and torques ***f*** ≡ ***τ***_1→*T*_ where

$$\begin{array}{ll} & \theta_{1\to T}=\left[\begin{array}{c} \theta_{1}\\ \vdots \\ \theta_{T} \end{array}\right],\;{\dot{\theta }}_{1\to T}=\left[\begin{array}{c} {\dot{\theta }}_{1}\\ \vdots \\ {\dot{\theta }}_{T} \end{array}\right],\\ & {\ddot{\theta }}_{1\to T}=\left[\begin{array}{c} {\ddot{\theta }}_{1}\\ \vdots \\ {\ddot{\theta }}_{T} \end{array}\right],\;\tau_{1\to T}=\left[\begin{array}{c} \tau_{1}\\ \vdots \\ \tau_{T} \end{array}\right], \end{array}$$

and the regressor matrix becomes

$$\Phi \left(Q\right)=\left[\begin{array}{c} \phi \left(\theta_{1},{\dot{\theta }}_{1},{\ddot{\theta }}_{1}\right)\\ \vdots \\ \phi \left(\theta_{T},{\dot{\theta }}_{T},{\ddot{\theta }}_{T}\right) \end{array}\right]$$

and (2) can be rewritten:

$$\Phi \left(Q\right)w=f.$$ (3)

Thus, the base parameters can be identified as the least-squares solution ***w*** ^⋆^:

$$w^{⋆}=\underset{w}{\mathrm{argmin}}\;\|\Phi w-f{\|}^{2}={\left(\Phi^{T}\Phi \right)}^{-1}\Phi^{T}f.$$ (4)

### 3.3 Optimization of robot reference trajectory

In practical applications, estimation ***w*** ^⋆^ is perturbed by errors due to noise-contaminated measurements. For example, the accuracy of the parameters is affected by the torque output errors *δ* ***f***, including the external disturbances.

The sensitivity of estimated parameters ***w*** ^⋆^ to the measurement errors can be evaluated by the condition number of the regressor matrix (Gautier and Khalil, 1992), which can be defined:

$$cond\left(\Phi \right)=\frac{\sigma_{\max}\left(\Phi \right)}{\sigma_{\min}\left(\Phi \right),}$$ (5)

where *σ*_*max*_(**Φ**) and *σ*_*min*_(**Φ**) denote the maximum and minimum singular values of the regressor matrix. If the condition number is small, the solution is insensitive to the errors. Therefore, it is important to obtain a wide variety of measurement data to reduce the condition number to reliably estimate the base parameters.

A conventional strategy to collect such measurement data is to first design a reference trajectory of robot $Q^{r}\equiv \left\{\theta_{1\to T}^{r},{\dot{\theta }}_{1\to T}^{r},{\ddot{\theta }}_{1\to T}^{r}\right\}$ by highly nonlinear optimization and generate its trajectory. The optimization problem can be formulated as:

$$Q^{⋆}=\underset{Q^{r}}{\mathrm{argmin}}\;cond\left(\Phi \right).$$ (6)

The derived movement $Q^{⋆}\equiv \left\{\theta_{1\to T}^{⋆},{\dot{\theta }}_{1\to T}^{⋆},{\ddot{\theta }}_{1\to T}^{⋆}\right\}$ is called the optimal robot excitation trajectory (Swevers et al., 1997).

To derive the optimal excitation trajectory for a humanoid robot, the constraints of balance must be considered so that the reference trajectory is generable by a humanoid robot without falling over. Even for an upper-limb humanoid robot, the balance constraints is important since the robot can fall over if the supporting base is not fixed to the ground.

The balance constraints are imposed on (6) by nonlinear inequality constraints:

$$h\left(Q^{r},w\right)\leq 0.$$ (7)

The calculation of the balance constraints requires base parameters ***w***. For example, the constraints of the Zero Moment Point (ZMP) are imposed to guarantee the robot’s balance (Bonnet et al., 2016). The ZMP can be represented:

$$p_{\mathrm{ZMP}}\left(\theta ,\dot{\theta },\ddot{\theta },w\right)=\left[\begin{array}{c} x-\frac{\left(z-l_{z}\right)\ddot{x}}{\ddot{z}+g}\\ y-\frac{\left(z-l_{z}\right)\ddot{y}}{\ddot{z}+g} \end{array}\right],$$ (8)

where (*x*, *y*, *z*) denotes the center of the mass position of the humanoid robot, and *l*_*z*_ and *g* are the height of the floor and the gravitational acceleration. The support polygon is a convex hull formed by the floor contact points. If the ZMP lies within the support polygon, the floor contact points do not change. Then, the robot can maintain the balance by using the ground reaction force from the contact points:

$$p_{\mathrm{ZMP}}^{-}\leq p_{\mathrm{ZMP}}\leq p_{\mathrm{ZMP}}^{+},$$ (9)

where we denote ZMP’s upper and lower boundaries as $p_{\mathrm{ZMP}}^{+,-}$ . Therefore, the left hand side of the inequality constraints in (7) can be formulated:

$$h\left(Q^{r},w\right)=\left[\begin{array}{c} h_{\mathrm{ZMP}}\left(\theta_{1},{\dot{\theta }}_{1},{\ddot{\theta }}_{1},w\right)\\ \vdots \\ h_{\mathrm{ZMP}}\left(\theta_{T},{\dot{\theta }}_{T},{\ddot{\theta }}_{T},w\right) \end{array}\right],$$

where

$$h_{\mathrm{ZMP}}\left(\theta ,\dot{\theta },\ddot{\theta },w\right)=\left[\begin{array}{c} p_{\mathrm{ZMP}}^{-}-p_{\mathrm{ZMP}}\\ p_{\mathrm{ZMP}}-p_{\mathrm{ZMP}}^{+} \end{array}\right].$$

However, the ZMP cannot be computed precisely if the initial model is unreliable. To account for inaccuracies, the conservative lower and upper bounds of the support polygon are considered instead of (9) in practice:

$$\left(1-\alpha \right)p_{\mathrm{ZMP}}^{-}\leq p_{\mathrm{ZMP}}\leq \left(1-\alpha \right)p_{\mathrm{ZMP}}^{+},$$ (10)

where *α* is the reduction coefficient of the support polygon (0 < *α* ≤ 1). As the reduction coefficient increases, the size of the support polygon is reduced. This restricts the variations of the reference trajectories, and the dynamics may not be fully excited. Therefore, a good initial model is necessary to estimate an accurate dynamics model.

The optimization (6) becomes more complex by enforcing the nonlinear inequality constraints (7). Deriving the reference trajectories can be intractable due to the large number of optimization variables and the nonlinear balance constraints.

## 4 Proposed method

Our identification method, on the other hand, requires neither preparing a good initial model nor solving the complex optimization problem. We construct a curriculum for system identification so that an accurate dynamics model is iteratively learned from an unreliable initial model. We select the reference trajectories from a robot motion dataset which is constructed using a large-scale human motion database. Our curriculum-based identification method is summarized in Algorithm 1. The details of our proposed method are described in this section.

Algorithm 1 Curriculum-Based Identification Algorithm.

 1: **Given**
 2:   Human motion dataset $\left(P_{1}^{h},P_{2}^{h},\ldots ,P_{K}^{h}\right)$

 3:   Initial base parameters ***w*** ^1^
 4:   Initial inequality constraints ***h*** ^1^
 5: **Run** $Motion\text{\_}retargeting\left(P_{1}^{h},\ldots ,P_{K}^{h}\right)$ :
 6:   **for** *k* = 1 **to** *K* **do**
 7:    Transfer $P_{k}^{h}$ into robot motions $Q_{k}^{r}$ by (15) and (16)
 8:   **end for**
 9:   **return** robot motion dataset $\left(Q_{1}^{r},\ldots ,Q_{K}^{r}\right)$

 10: **Run** $Iterative\text{\_}identification\left(w^{1},h^{1},Q_{1}^{r},\ldots ,Q_{K}^{r}\right)$ :
 11:   **for** *i* = 1 **to** *I* **do**
 12:    Select reference ***Q*** ^⋆^ from $\left(Q_{1}^{r},\ldots ,Q_{K}^{r}\right)$ by (11)
 13:    Sample measurement data ***Q*** ^*i*^ and ***f*** ^*i*^ by using ***Q*** ^⋆^
 14:    Compute new base parameters ***w*** ^*i*+1^ by (4)
 15:    Construct new inequality constraints ***h*** ^*i*+1^
 16:   **end for**
 17:   **return** estimated parameters ***w*** ^*I*+1^

### 4.1 Iterative identification

We define a curriculum as a series of robot reference trajectories. By sequentially generating the reference trajectories by the robot, a wider variety of measurement data can be sampled. Consequently, an accurate model can be learned gradually without a good initial model. To perform this process, we developed an iterative identification method (lines 10 to 17 of Algorithm 1). Our method repeats an identification process until the iteration reaches the maximum number of iterations: *I*.

At each iteration, we obtain reference trajectory ***Q*** ^⋆^ (line 12 of Algorithm 1). In *i*-th iteration, the reference trajectory is selected from a dataset of *K* robot motions $\left(Q_{1}^{r},Q_{2}^{r},\ldots ,Q_{K}^{r}\right)$ :

$$Q^{⋆}=\underset{Q^{r}\in \left(Q_{1}^{r},\ldots ,Q_{K}^{r}\right)}{\mathrm{argmin}}\;cond\left(\Phi \right),$$ (11)

such that

$$h^{i}\left(Q^{r},w^{i}\right)\leq 0.$$ (12)

Since this simple optimization avoids solving the complex optimization problem (6, 7), a wide variety of reference trajectories can be easily obtained.

Note that in our proposed method, the robot motion dataset for iterative identification was constructed by converting the captured human data into robot motion, i.e., motion retargeting. Due to the similarity of the structure to humans, the motion data for a humanoid robot can be acquired by capturing a human movement (Yamane, 2011; Ayusawa and Yoshida, 2017). Thus, we are able to obtain a sufficient amount of motion data for robots from a large-scale human motion dataset. The retargeting process is described in Section 4.2.

In the optimization (11), we consider a concatenated regressor matrix with all the measurement data that were sampled in the previous iterations from 1 to *i* − 1 and the newly selected reference trajectory in current iteration *i*:

$$\Phi \leftarrow \left[\begin{array}{c} \Phi \left(Q^{1}\right)\\ \Phi \left(Q^{2}\right)\\ \vdots \\ \Phi \left(Q^{i-1}\right)\\ \Phi \left(Q^{r}\right) \end{array}\right].$$

This enables us to find an effective reference trajectory to estimate the unreliable base parameters that were not excited by the previously obtained measurement data.

**FIGURE 2:**
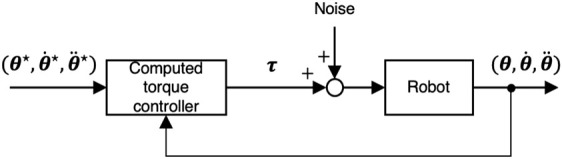
Data sampling process: Each data point of reference trajectories $\left(\theta^{⋆},{\dot{\theta }}^{⋆},{\ddot{\theta }}^{⋆}\right)$ is generated by robot to obtain data $\left(\theta ,\dot{\theta },\ddot{\theta },\tau \right)$ for identification. Noise input was added to torque command to simulate torque output errors.

By reproducing the reference trajectory on the robot, the measurement data of $Q^{i}\equiv \left\{\theta_{1\to T}^{i},{\dot{\theta }}_{1\to T}^{i},{\ddot{\theta }}_{1\to T}^{i}\right\}$ , and $f^{i}\equiv \tau_{1\to T}^{i}$ are obtained (line 13 of Algorithm 1). In this paper, we utilized a computed torque controller to generate reference motion (Khosla and Kanade, 1989):

$$\tau_{t}=\phi \left(\theta_{t}^{⋆},{\dot{\theta }}_{t}^{⋆},{\ddot{\theta }}_{t}^{⋆}\right)w+K_{p}\left(\theta_{t}^{⋆}-\theta_{t}\right)+K_{d}\left({\dot{\theta }}_{t}^{⋆}-{\dot{\theta }}_{t}\right),$$ (13)

where ***K***_*p*_ and ***K***_*d*_ denote the position and velocity gains. These gains can be small in the computed torque controller since we use an inverse dynamics controller. Figure 2 shows a block diagram of the data sampling. We investigate the noise perturbation of the joint torques.

After sampling the new measurements, we stack the regressor matrix and the torque sequence with the previously obtained measurements:

$$\Phi \leftarrow \left[\begin{array}{c} \Phi \left(Q^{1}\right)\\ \Phi \left(Q^{2}\right)\\ \vdots \\ \Phi \left(Q^{i-1}\right)\\ \Phi \left(Q^{i}\right) \end{array}\right],\;f\leftarrow \left[\begin{array}{c} f^{1}\\ f^{2}\\ \vdots \\ f^{i-1}\\ f^{i} \end{array}\right],$$

and new parameters ***w*** ^*i*+1^ are estimated using (4) (line 14 of Algorithm 1).

Finally, we construct new inequality constraints ***h*** ^*i*+1^ for next iteration *i* + 1 (line 15 of Algorithm 1). At the first iteration, initial parameters ***w*** ^1^ are unreliable. Thus, initial inequality constraints ***h*** ^1^ restrict the reference trajectories to be generable with the unreliable dynamics model. In many instances, quasi-static motions are selected in the first iteration. But, as the iteration proceeds, the robot might generate more aggressive motions than the previous iterations since the parameters are iteratively updated. Therefore, we relax the inequality constraints at each iteration so that more dynamic reference trajectories can be selected in the optimization (11).

### 4.2 Motion retargeting

Before initiating the iterative identification process, we transformed the human motion data into those of the robot (lines 5 to 9 of Algorithm 1) in a process called motion retargeting (Gleicher, 1998).

We denote a sequence of three-dimensional positions of markers measured by a motion capture system as ***p*** ^*m*^. We define the human motion data with *T* time steps as $P^{m}\equiv \left\{p_{1}^{m},p_{2}^{m},\ldots ,p_{T}^{m}\right\}$ . In a human motion database, multiple human motion data are contained. The first process’s goal is to transform each human motion bit of data ***P*** ^*m*^ into robot motion data ***Q*** ^*r*^.

We achieve this by first defining the kinematics model for representing the human motion data. Next we compute geometric parameters ***ξ*** ^*h*^ and angle trajectories $\theta_{1\to T}^{h}$ of the human model so that the sequence of the marker positions of human model $P^{h}\equiv \left\{p_{1}^{h}\left(\xi^{h},\theta_{1}^{h}\right),p_{2}^{h}\left(\xi^{h},\theta_{2}^{h}\right),\ldots ,p_{T}^{h}\left(\xi^{h},\theta_{T}^{h}\right)\right\}$ is close to that of the captured data. Moreover, we compute robot angle trajectories $\theta_{1\to T}^{r}$ so that the sequence of the marker positions of robot $P^{r}\equiv \left\{p_{1}^{r}\left(\theta_{1}^{r}\right),p_{2}^{r}\left(\theta_{2}^{r}\right),\ldots ,p_{T}^{r}\left(\theta_{T}^{r}\right)\right\}$ is close to that of the human model. This can be done by a motion retargeting method (Ayusawa and Yoshida, 2017) that directly optimizes geometric parameters ***ξ*** ^*h*^ and joint angles $\theta_{1\to T}^{h}$ and $\theta_{1\to T}^{r}$ . However, such a choice entails huge computation cost since a large amount of motion data must be retargeted in our approach. To avoid this problem, we used the B-spline function to reduce the size of the optimization problem (Rackl et al., 2012).

We respectively define the *N* control points equally spaced between *t* = 1 to *T* (assuming *N* < *T*) and their corresponding basis functions of the B-spline function:

$$\theta_{1\to N}^{s,h}\equiv \left[\begin{array}{c} \theta_{1}^{s,h}\\ \vdots \\ \theta_{N}^{s,h} \end{array}\right],\;\theta_{1\to N}^{s,r}\equiv \left[\begin{array}{c} \theta_{1}^{s,r}\\ \vdots \\ \theta_{N}^{s,r} \end{array}\right],\;b_{1\to N}^{s}\equiv \left[\begin{array}{c} b_{1}^{s}\\ \vdots \\ b_{N}^{s} \end{array}\right],$$

where $\theta_{1\to N}^{s,h}$ denotes the series of angle trajectories of the human model corresponding to *N* control points and $\theta_{1\to N}^{s,r}$ denotes the series of robot angle trajectories corresponding to the control points.

Then joint angles $\theta_{1\to T}^{h}$ and $\theta_{1\to T}^{r}$ can be computed:

$$\theta_{t}^{h}=\overset{N}{\underset{j=1}{\sum }}\theta_{j}^{s,h}b_{j}^{s}\left(t\right),\;\theta_{t}^{r}=\overset{N}{\underset{j=1}{\sum }}\theta_{j}^{s,r}b_{j}^{s}\left(t\right).$$ (14)

By using (14), we formulate motion retargeting as the following optimization problem:

$$\begin{array}{ll} & \underset{\left(\xi^{h},\theta_{1\to T}^{s,h},\theta_{1\to T}^{s,r}\right)}{\min}\left\{\|P^{h}\left(\xi^{h},\theta_{1\to N}^{s,h}\right)-P^{m}{\|}^{2}+w_{g}\|P^{r}\left(\theta_{1\to N}^{s,r}\right)\right.\\ & \;\left.-P^{h}\left(\xi^{h},\theta_{1\to N}^{s,h}\right){\|}^{2}+w_{\eta }\eta \left(\theta_{1\to N}^{s,r}\right)\right\}, \end{array}$$ (15)

where *η* is a penalty function and *w*_*η*_ is its weight. In this paper, *η* was used to constrain the joint angles so that they do not exceed their limitations. Moreover, *w*_*g*_ is a weight corresponding to the series of relative position errors of the markers between the human model and the robot.

The computational cost of Algorithm 1 is dominated by the optimization process for motion retargeting. We adopted the quasi-Newton algorithm implemented as a Matlab function for solving the retargeting problem in (15), where the computational complexity of the quasi-Newton method is known as *O*(*n* ^2^). Since we used the B-spline functions to represent the movements, knot points were variables to be optimized. The number of optimized variables for each iterative computation was *n* = *n*_*ξ*_ + 2*Nn*_*θ*_, where *n*_*ξ*_ is the number of geometric parameters ***ξ*** ^*h*^, and *n*_*θ*_ denotes the number of joint angles ***θ*** ^*h*^ or ***θ*** ^*r*^.

The derivatives of the angle trajectories are calculated using the finite difference method:

$$\left({\dot{\theta }}_{t}^{r},{\ddot{\theta }}_{t}^{r}\right)=\left\{\begin{array}{cc} \left(0,0\right)\; & \text{if }t=1\\ \left(\frac{\theta_{t}^{r}-\theta_{t-1}^{r}}{\Delta t},\frac{{\dot{\theta }}_{t}^{r}-{\dot{\theta }}_{t-1}^{r}}{\Delta t}\right)\; & \text{otherwise}, \end{array}\right.$$ (16)

where Δ*t* denotes the time step of motion retargeting.

## 5 Experiments

We conducted experiments on the simulated environment to evaluate our curriculum-based identification approach. The details of the experimental setups and results are provided in this section.

### 5.1 Evaluation setups

We first verified that our method could identify a robot’s accurate dynamical model without a good initial model. We did 200 identification experiments. For each identification, different sequences of zero-mean Gaussian noise *δ* ***f*** were added to the computed torque (Figure 2) to simulate torque output errors in the identification results. The standard deviation of the noise was set to 5% of the torque limits (Presse and Gautier, 1993). The maximum number of iterations of our iterative identification method was set to *I* = 5 so that the identification process might not be finished before the accurate model was obtained.

We prepared an unreliable initial model and examined whether the estimation error of the base parameters was gradually reduced as the learning iterations proceeded. The estimation error is measured by

$$RMSE_{w}=\sqrt{\frac{1}{D}\|w^{\mathrm{true}}-w{\|}^{2}},$$ (17)

where ***w*** ^*true*^ is the true base parameter vector and *D* is the dimension of the base parameter vector, i.e., the number of parameters. (The relevant results are shown in Figure 6.)

The reliability of the parameters was evaluated using the relative standard deviation of each estimated parameter:

$$d_{w_{j}\%}=100\frac{d_{e,j}}{\left|{\hat{w}}_{j}\right|},$$ (18)

where ${\hat{w}}_{j}$ denotes the *j*-th element of the mean value of 200 estimated base parameters and *d*_*e*,*j*_ is the standard deviation of its estimation error. A parameter is regarded as well estimated if $d_{w_{j}\%}$ is within 10% or ${\hat{w}}_{j}$ is small (<0.02) (Ayusawa et al., 2009).

The reliability was also evaluated by validation. We prepared reference motion trajectories for validation and obtained measurement data $\left(\theta_{t}^{c},{\dot{\theta }}_{t}^{c},{\ddot{\theta }}_{t}^{c},\tau_{t}^{c}\right)$ using the reference motion trajectories and the true base parameters by (13). The root-mean square errors (RMSE) of joint torques (Ogawa et al., 2014) for the initial and estimated base parameters ***w*** were calculated by

$$RMSE_{\tau }=\sqrt{\frac{1}{T}\overset{T}{\underset{t=1}{\sum }}\|\tau_{t}^{c}-\phi \left(\theta_{t}^{c},{\dot{\theta }}_{t}^{c},{\ddot{\theta }}_{t}^{c}\right)w{\|}^{2}}.$$ (19)

(The relevant results is shown in Figure 8.)

We next evaluated our simple optimization (11). Our proposed method created a curriculum by selecting appropriate reference trajectories from the robot motion dataset. We compared our proposed method with a non-curriculum-based approach that did not solve the optimization problem (11). This approach randomly picked a reference trajectory under the constraints (12). We examined whether the base parameters can be learned efficiently if the sampling process was guided by the curriculum (The relevant results are shown in Figure 7).

In addition, we also evaluated our curriculum by comparing the obtained model with the other models which were identified by using single motions. Here, we called the former “the non-task-specific model” and the latter “the task-specific models.” At first, we identified the task-specific models by using each of the two groups of motions (Open and Scoop), which could be selected in the first iteration of the identification curriculum in Figure 4. After identifying each model, we evaluated the reliability of the model by using (19). (The relevant results are shown in Figure 8).

Moreover, we evaluated the efficiency of using B-spline functions for motion retargeting (Section 4.2). To compare with using B-spline functions, we prepared another strategy which directly optimized the joint angles $\theta_{1\to T}^{h}$ and $\theta_{1\to T}^{r}$ without using the B-spline function (14). We performed the motion retargeting 10 times with each strategy by using a sampled motion (*T* = 740). After retargeting, for each strategy, we evaluated the computation time and the optimization costs (The relevant results are shown in Figures 9A, B).

### 5.2 Experimental setups

#### 5.2.1 Robot model

We conducted simulated experiments using an upper-limb humanoid robot model of *Torobo* (Tokyo Robotics Inc.) to evaluate our proposed method. Assuming a risk of falling over, we investigated the balance constraints using ZMP. The robot has a total 18 DoFs; each arm has 7 DoFs, its torso has 2 DoFs, and its head has 2 DoFs.

A dynamic simulation was performed with a physics engine *MuJoCo* (Todorov et al., 2012). The sensor information of each joint angle (and its derivatives) and torque were measured at 10-ms intervals. Gains ***K***_*p*_ and ***K***_*d*_ for the proportional derivative (PD) controller in (13) were manually designed. Note that the robot could not follow the reference trajectories by the PD controller since we only set the gains to be small.

The robot has a total of *D* = 132 identifiable base parameters. In the experiment, we regarded the CAD parameters as the true inertial parameters ***w*** ′^*true*^ to derive true base parameters ***w*** ^*true*^. Inertial parameters ***w*** ′^1^ for deriving initial base parameters ***w*** ^1^ were randomly generated from a uniform distribution. If the *j*-th element of ***w*** ′^*true*^ exceeds 0, the uniform distribution interval was $\left[0,2w_{j}^{\prime true}\right]$ ; otherwise it was $\left[2w_{j}^{\prime true},0\right]$ .

#### 5.2.2 Relaxation of inequality constraints

At each iteration of our identification method, we relaxed the inequality constraints (12) by computing a reduction coefficient of the support polygon at each iteration by

$$\alpha_{\mathrm{iter}}=a\left\{cond\left(\Phi \right)-b\right\},$$

where reduction coefficient *α* in (10) is decreased by the following criteria:

$$\alpha =\left\{\begin{array}{cc} \alpha_{\mathrm{iter}}\; & \left(\text{if }\alpha_{\mathrm{iter}}<\alpha_{\max}\right)\\ \alpha_{\max}\; & \left(\text{otherwise}\right), \end{array}\right.$$ (20)

where *α*_*max*_, *a*, and *b* respectively denote the maximum reduction coefficient, scaling, and bias factors. In the first iteration, *α* was initialized with *α*_*max*_. We set *α*_*max*_ = 0.74 so that only 5% of the reference trajectories could be selected from the robot motion dataset at the first iteration. We used *a* = 2.5 × 10^−4^ and *b* = 1.0 so that *α* did not become too small value in the initial iteration.

Since condition number *cond*(**Φ**) might decrease as the iteration proceeds, reduction coefficient *α* was also decreased. Therefore, the support polygon gradually expanded, and our proposed method selected more aggressive motions as learning progressed.

#### 5.2.3 Human motion database and setups for retargeting

We utilized for motion retargeting the *KIT Bimanual Manipulation Dataset*(Krebs et al., 2021), which contains a large number of capture data of a human’s bimanual daily household activities, e.g., opening and closing lids, wiping dishes, etc. Each captured motion was stored in the Coordinate 3D (C3D) format, which recorded the three-dimensional coordinates of each part of the human body at 100 Hz sampling frequency (Δ*t* = 10 ms period). We took 110 motions from the dataset, 98 of which were used as a reference motion dataset for identification, and 12 of which were used for validation.

The motion retargeting was performed using the *minFunc* function (Schmidt, 2005) (which had been made for solving optimization problems) in a Matlab environment by an AMD EPYC 7742 CPU, 3.4-GHz computer. The weights of the penalty function were set to *w*_*g*_ = 100 and *w*_*η*_ = 50. The angle trajectories were generated by interpolating the via points, which were optimized at 200-ms intervals. After retargeting, each bit of data was labeled by the motion names shown in Table 1, where the names were taken from the used dataset.

**TABLE 1:** Motion dataset (Krebs et al., 2021) used for system identification and validation.

Motion name	Amount of data	Duration
Close	8	5.29–9.25 s
Cut	7	7.65–11.41 s
Mix	6	7.41–8.33 s
Open	6	5.89–7.65 s
Peel	6	7.81–12.13 s
Pour	14	5.41–9.85 s
Rollout	8	7.69–11.21 s
Scoop	16	5.93–8.33 s
Stir	16	3.81–10.69 s
Sweep	7	10.29–13.17 s
Transfer	8	5.53–6.81 s
Wipe	8	5.69–8.73 s

### 5.3 Experimental result

**FIGURE 3:**
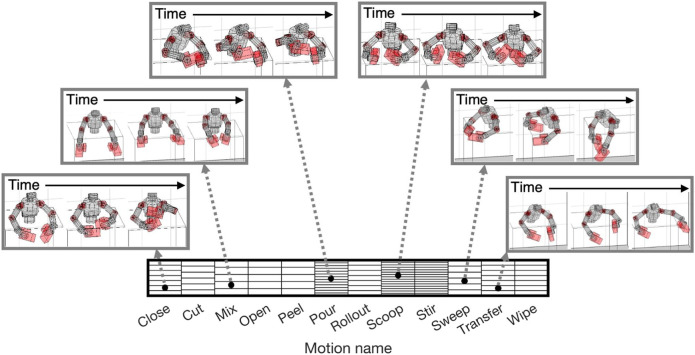
Reference motion dataset for upper-limb humanoid robot: Each block represents reference motion trajectory. Trajectories were sorted according to labels of motion names. By retargeting large-scale human motion database, we contained a wide variety of motion trajectories. Snapshots of some motions are shown as examples.

Our proposed method selected a sequence of the reference trajectories from the robot motion dataset to create a curriculum. Figure 3 shows the robot motion dataset. Since we constructed it using a large-scale human motion database, it contains various motion trajectories (e.g., Figure 3).

**FIGURE 4:**
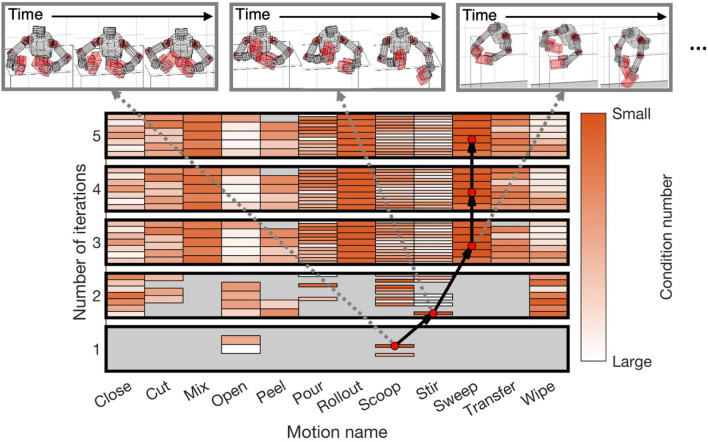
Identification curriculum for upper-limb humanoid robot: Each rectangle enclosed by thick black line represents generable reference motion dataset for each iteration. Color of each block represents relative magnitude of condition number for each iteration. Here, darker color represents smaller condition number than lighter color. Black arrows represent curriculum which connects motion with smallest condition number (red dots) at each iteration.

**FIGURE 5:**
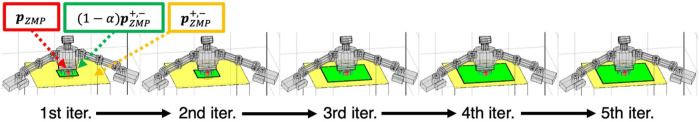
Relaxation of balance constraints for each iteration of representative curriculum: Red dot (***p***_*ZMP*_) represents ZMP, computed using reference trajectory. Yellow rectangle $\left(p_{\mathrm{ZMP}}^{+,-}\right)$ represents upper and lower boundaries of support polygon. Green rectangle $\left(\left(1-\alpha \right)p_{\mathrm{ZMP}}^{+,-}\right)$ represents reduced lower and upper bounds of support polygon by (20). Reduction coefficient *α* was taken as 0.74 at first iteration and reached 0.3 at fifth iteration. Therefore, size of support polygon (green rectangle) becomes larger as iterations proceeded.

By selecting appropriate trajectories from the dataset, 200 learning curricula were created in the experiments because we performed identification experiments 200 times in different noise settings. A representative curriculum is depicted in Figure 4. At each iteration, the reference trajectory with the minimum condition number was selected from the entire dataset. The varieties of reference trajectories increased as iterations progressed since the upper and lower boundaries of the ZMP were expanded with (20) (Figure 5). For example, the sweep motions were unavailable in the first and second iterations, although they became generable from the third iteration and had the smallest condition numbers.

**FIGURE 6:**
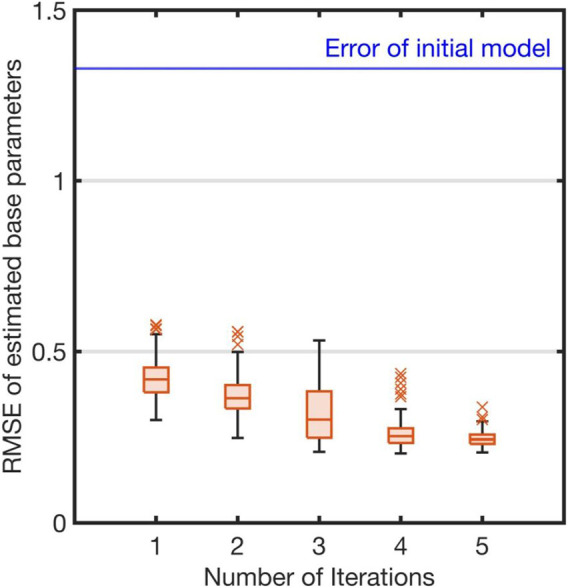
Estimation errors of base parameters: Blue line represents root mean square error (RMSE) between true and initial parameters of robot model. Each red box represents RMSEs between true and estimated parameters of each iteration. The red crosses represent the outliers in each iteration of the evaluation using the proposed method.

Figure 6 shows the RMSE between the true and estimated parameters (17) for each iteration of the identification. The error of the initial model is indicated with a solid line. The estimation errors gradually reduced as iterations proceeded (Figure 6). Finally, the error median was 0.243 at the fifth iteration. We evaluated the reliability of the parameters by relative standard deviation (18). Our proposed method successfully estimated 75.8% of the parameters (100 of 132) at the fifth iteration. In the initial model, 57.6% of the parameters (76 of 132) were reliable. Therefore, our method learned an accurate dynamics model where a good initial model was unavailable.

**FIGURE 7:**
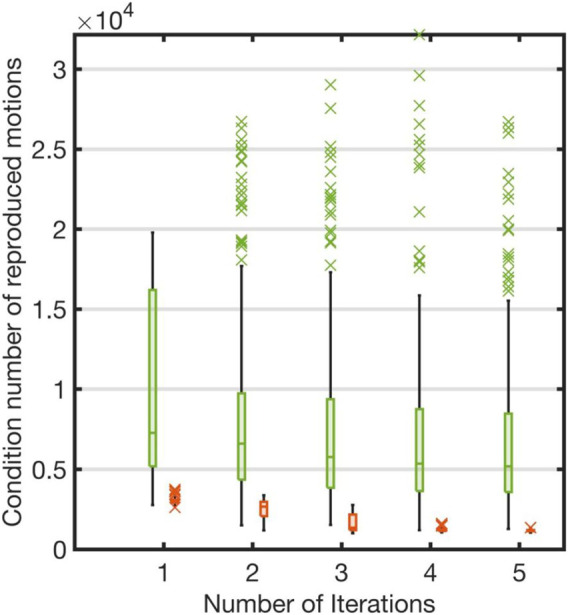
Comparison of curriculum-based and non-curriculum-based approaches: Each red box represents condition numbers of our curriculum-based approach. Each green box represents condition numbers of non-curriculum-based approach which randomly picked reference motions for each iteration. The red crosses represent the outliers in each iteration of the evaluation using the proposed method. The green crosses represent the outliers using non-curriculum-based approach in each iteration.

Figure 7 compares our curriculum-based and the non-curriculum-based approaches. As shown in Figure 7, the condition number was significantly large at each iteration if the reference trajectories were selected randomly from the robot motion database. Our curriculum-based approach, on the other hand, efficiently learned the accurate base parameters. Therefore, the results showed the importance of guiding the sampling process by creating the curriculum.

**FIGURE 8:**
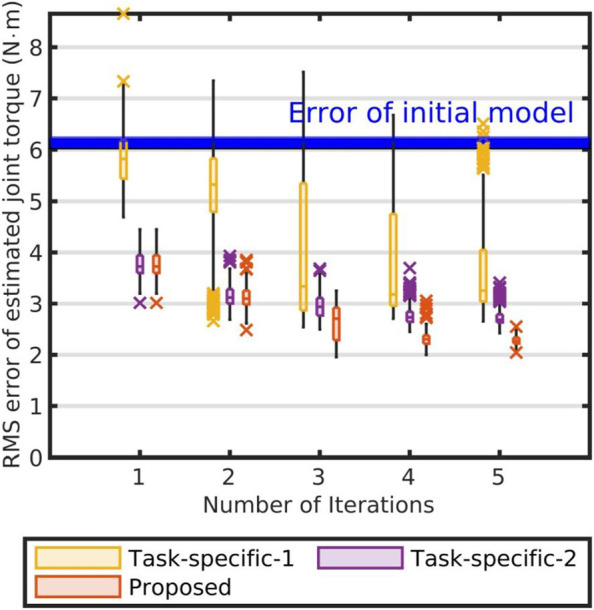
Comparison of task-specific and non-task-specific models: Blue band represents RMSEs between estimated joint torques using initial base parameters and their measurements. Each box represents RMSEs between estimated joint torques using identified parameters of each iteration and their measurements. Each yellow box represents RMSEs between measurements and estimations using identified base parameters by leveraging only “Open” motions. Each violet box represents RMSEs between measurements and estimations using identified base parameters by leveraging only “Scoop” motions. Each red box represents RMSEs between measurements and estimations using base parameters of the non-task-specific model. The crosses represent the outliers in each iteration of the evaluation.

Figure 8 compare the non-task-specific model and the task-specific models. As shown in Figure 8, in case of the non-task-specific model, the errors became smaller than that of the task-specific models for each iteration. Therefore, our curriculum estimated more reliable model than the task-specific models.

**FIGURE 9:**
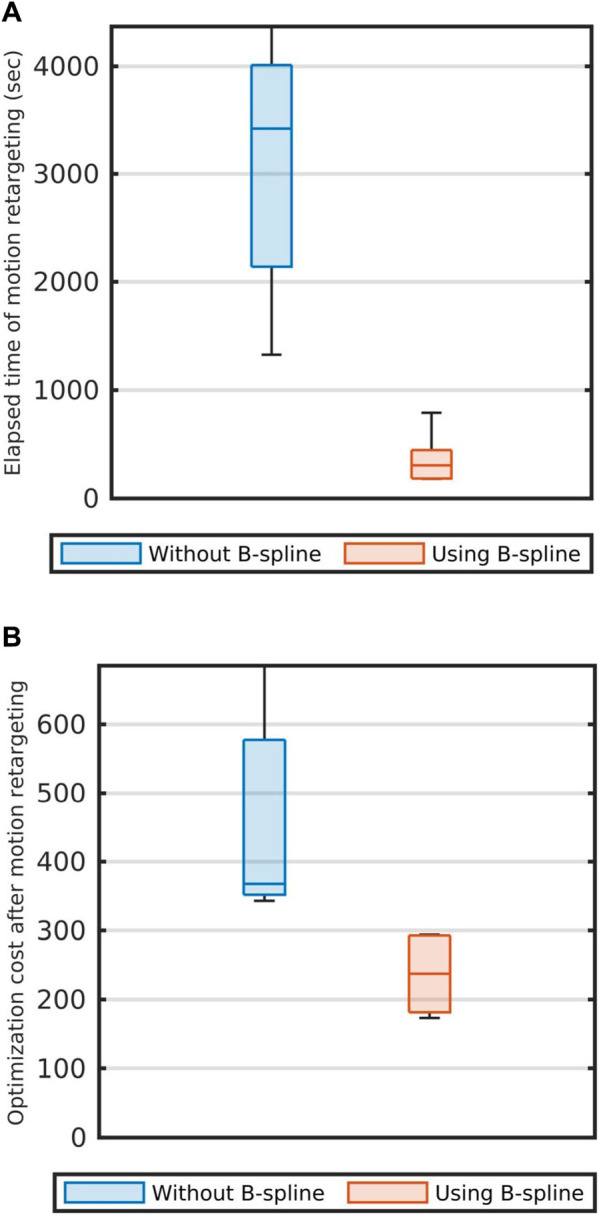
Comparison of the strategies of the motion retargeting. **(A)** The calculation times which were taken for motion retargeting. **(B)** the optimization costs. In both subfigures, blue boxes represent the results when motion retargeting is performed without using the B-Spline function. Red boxes represent the results when motion retargeting is performed using the B-Spline function.

Figures 9A, B compare the performances of the motion retargeting: The blue boxes show the performance of retargeting without B-spline and the red boxes show the performance of retargeting with using B-spline. As shown in Figures 9A, B, when optimization was performed using (14), we could obtain results with lower optimization costs and less computation time than when B-spline functions were not used. Therefore, motion retargeting using the B-spline functions was shown to be more efficient.

## 6 Conclusion

We proposed a curriculum-based approach for the system identification of a humanoid robot. Since the curriculum helps gradually learn accurate dynamics from an unreliable initial model, our method does not require a good initial model. Unlike the conventional approach, our method does not need to solve the complex optimization problem. By using a large-scale human motion database, various reference motions can be provided by converting the human motion data into those of the robot. By selecting an appropriate reference trajectory, our curriculum successfully guided the robot’s sampling process. Consequently, an accurate dynamics model was gradually learned from an unreliable initial model by our iterative identification method.

Future work will apply our curriculum-based identification method to a physical robot. In addition, we will evaluate our approach on a whole-body humanoid robot. In this case, we would not only change the robot model, but also consider whether there are other motion databases which would be suitable for identifying the model. Once the accurate initial model is obtained, we would try to identify more accurate model for the fast and precise control. Then, it is necessary to consider whether it is more preferable to carry out our identification curriculum using a database containing more aggressive behaviors or to use a conventional method without using a database.

Moreover, we will verify the end conditions of iterative identifications in the future work. In this paper, the maximum number of iterations was set in advance to demonstrate the effectiveness of the identification curriculum. However, it should be determined to ensure that a highly accurate model is obtained. To do so, some criteria could be used as the end conditions of identification [e.g., the condition number of the angle trajectories (5) and the reliability of the model (19)]. Then, the tuning of the hyperparameters for the relaxation of the inequality constraint might also be discussed to avoid generating a redundant curriculum.

## Data Availability

The datasets presented in this article are not readily available because the data is not open to the public. Requests to access the datasets should be directed to kang@atr.jp.
